# Heme iron polypeptide for the treatment of iron deficiency anemia in non-dialysis chronic kidney disease patients: a randomized controlled trial

**DOI:** 10.1186/1471-2369-14-64

**Published:** 2013-03-20

**Authors:** Shankar P Nagaraju, Adam Cohn, Ayub Akbari, Janet L Davis, Deborah L Zimmerman

**Affiliations:** 1 Department of Nephrology, Kasturba Medical College, Manipal, Karnataka, India; 2 Department of Medicine, Queensway Carleton Hospital, Ottawa, ON, Canada; 3 Department of Medicine, Division of Nephrology, Ottawa Hospital Research Institute, Ottawa, ON, Canada; 4 Department of Medicine, Division of Nephrology, Ottawa, ON, Canada

**Keywords:** Anemia, Iron, Chronic kidney disease

## Abstract

**Background:**

Anemia secondary to iron deficiency is common in patients with non-dialysis dependent chronic kidney disease (ND-CKD) but it is unclear if oral supplementation is as effective as intravenous (IV) supplementation in re-establishing iron stores. The purpose of this study was to determine if oral Heme Iron Polypeptide (HIP) is as effective as IV iron sucrose in the treatment of iron-deficiency anemia for patients with ND-CKD.

**Methods:**

Forty ND-CKD patients were randomized; 18 to HIP 11 mg orally 3 times per day and 22 to IV iron sucrose 200 mg monthly for 6 months. Baseline clinical and laboratory data were collected for all patients. The primary and secondary outcomes for the study were hemoglobin (Hgb) concentration and iron indices [ferritin and percentage transferrin saturation (TSAT)] at the end of 6 months respectively. Adverse events were also compared.

**Results:**

The baseline demographic characteristics and laboratory values were similar for the two groups. After 6 months of treatment, Hb in the HIP group was 117 g/L and 113 g/L in the IV sucrose group (p = 0.37). The TSAT at 6 months was not different between the two groups {p = 0.82}but the serum ferritin was significantly higher in the IV iron sucrose group {85.5 ug/L in HIP and 244 ug/L; p = 0.004}. Overall adverse events were not different between the groups.

**Conclusion:**

HIP is similar in efficacy to IV iron sucrose in maintaining hemoglobin in ND-CKD patients with no differences in adverse events over 6 months. It is unclear if the greater ferritin values in the IV iron sucrose group are clinically significant.

**Trial registration:**

ClinicalTrials.gov: NCT00318812

## Background

Anemia develops early during chronic kidney disease (CKD) and is associated with increased cardiovascular morbidity, mortality and decreased quality of life [1-3]. Iron deficiency is common in patients with CKD which limits the effectiveness of erythropoiesis-stimulating agents (ESA) [4-7]. The estimated prevalence of iron deficiency ranges from 25 to 70% [8,9]. Importantly iron has many other physiologic functions that may be important for overall health such as immune function, thermoregulatory performance, energy metabolism, and exercise or work performance [10].

To replete iron stores, iron can be administered either orally or intravenously (IV). Although oral iron is less expensive, easier to administer, and may be safer, IV iron enables the administration of larger doses of iron and is better tolerated by some patients [11]. The main adverse reactions to oral iron are gastrointestinal and may limit adherence and dose [12]. The most feared adverse reaction to IV iron is anaphylaxis, which is more common with iron dextran than with other preparations [13,14]. In addition, there are concerns that IV iron may accelerate kidney damage in patients with CKD not on dialysis therapy, promote infections by supplying iron to pathogenic bacteria, enhance atherosclerosis by generating oxidative stress, and cause endothelial damage [15-18].

In hemodialysis patients several different studies, including a randomized controlled trial, have consistently demonstrated that intravenous iron supplementation is superior to oral iron replacement with respect to enhancing body iron stores, augmenting hemoglobin levels and reducing ESA requirements [19-21]. Among patients with ND-CKD, by contrast, evidence for an optimal iron replacement strategy, safety, and test utility is less clear. There is controversy about when to start iron supplementation, target values for ferritin and TSAT such that the 2008 Canadian Society of Nephrology guidelines for management of iron deficiency anemia are Grade D [22]. There is also ongoing controversy as to whether iron supplementation is best administered orally or intravenously in ND-CKD and peritoneal dialysis patients [23]. There are only a few comparative studies between IV iron infusion and oral iron supplementation and the conclusions are conflicting [24-28].

Heme iron polypeptide (HIP) is a new generation oral iron which uses the heme porphyrin ring to supply iron to sites of absorption in the intestinal lumen. In comparison with the other standard iron preparations, preliminary evidence suggests that HIP may represent a promising new strategy for oral iron replacement [29-31]. For this reason, we performed a randomized controlled trial to determine if oral HIP is as effective as IV iron sucrose in the treatment of iron-deficiency anemia for patients with ND-CKD.

## Methods

### Study design

This was a single blind (investigator), randomized controlled trial performed at the Ottawa hospital from May 2007 to February 2011(NCT00318812). Randomization was via a computer generated sequence; group allocation was stored in sealed opaque sequentially numbered envelopes. The study protocol and all amendments were approved by the Ottawa Hospital Research Ethics Board. The original protocol was to include patients with an eGFR < 30 mls/minute and Hgb of 90–110 but was modified secondary to recruitment challenges. All ND-CKD patients > 18 years old with an estimated glomerular filtration rate (eGFR) ≤60 ml/min/1.73 m^2^ with anemia [90–120 g/L (females) 90–135 g/L (males)] and iron indices lower than the CSN recommended targets (serum ferritin < 100 ucg/L or TSAT < 20%) were invited to participate. Patients were excluded if they had received parenteral iron therapy or blood transfusion within the last 3 months, were pregnant, or had a history of recent malignancy, infection, GI bleed or major surgery. If the patient was already on an oral iron preparation, the preparation was stopped and the patient was included in the study after a wash out period of two weeks. Patients were also excluded if serum folate or vitamin B12 levels were below the normal limits (< 15 nmol/L, <133 pmol/L respectively). If the participant was being treated with an ESA, the medication was continued and the dose was adjusted by the blinded study investigator to maintain Hgb from 100-120 g/L. If the participant was not on an ESA at study entry, once the participant was iron replete (TSAT 20-50% and ferritin 100-500 ucg/L), if the Hb was <100 g/L, an ESA was started.

### Measurements

Laboratory tests were done at the Ottawa Hospital. Hemoglobin was measured from plasma using spectrophotometric scan with a coefficient of variation of 4%. Ferritin was measured with an automated immunoassay with a CV of 5%. Iron and transferrin were measured with the ferrozine method with a CV of 3% and immunonephelometry with a CV of 5% respectively.

### Treatment and follow up

After providing informed consent, patients were randomized to receive IV iron sucrose 200 mg monthly or HIP 11 mg orally three times a day for total of 6 months. Absorption of HIP in chronic kidney disease is approximately 18.6% such that 33 mg per day is roughly equivalent to 200 mg IV iron sucrose [32].

Baseline clinical data and laboratory investigations were collected as per the protocol at the time of enrollment. Patients were followed monthly for compliance (pill counts) and possible adverse effects (standardized questionnaire) in both groups. The questionnaire specifically asked patients to quantify (none, somewhat/occasionally, a lot/often) if they experienced constipation (<1 bowel movement per 2 days), diarrhea (> 3 bowel movements per day), bloating, nausea, cramps, indigestion, muscle cramps, episodes of low blood pressure and skin rash at 2, 4 and 6 months. Hemoglobin was repeated monthly and iron indices were repeated every two months.

Premature withdrawal was defined as initiation of renal replacement therapy, blood transfusion, non-adherence (refusal to take study medication) or withdrawal of consent.

### Outcome

Summary descriptive statistics were calculated to describe the study patient population using SAS enterprise (version 4.2). Results are expressed as median and interquartile range for continuous data and percentage and frequency for categorical data. The primary outcome was a comparison of Hb concentration at 6 months between the two groups using the Wilcoxon two –sample test. A similar analysis was done for the secondary outcome measures (serum ferritin, TSAT). Intent to treat analysis was utilized. Where there was missing data, the last value was carried forward for any patients with a premature withdrawal from the study protocol for whatever reason. We also examined the requirement for erythropoietin and adverse events with the medication.

## Results

**Figure 1:**
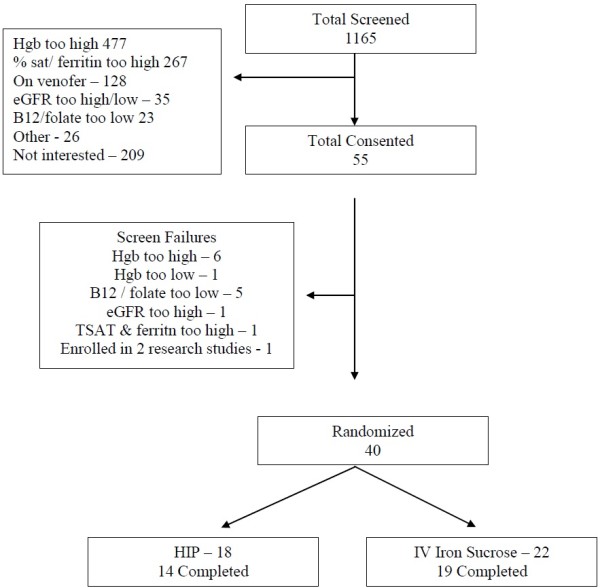
Trial flow.

Between May 2007 to February 2011, 55 patients were consented to participate in the study and 40 patients were randomized to the treatment groups (Figure 1). There were 22 patients in the IV iron sucrose group and 18 patients in the oral HIP group. Demographics and baseline characteristics of the study population were similar in the two groups as shown in Table 1.

**Table 1:** Baseline characteristics of both groups (median/interquartile range)

**Parameter**	**HIP**	**IV sucrose**	**p-value**
Number of patients (Randomized)	18	22	
Age(years)	76 (66–83)	66 (58–76)	0.10
Sex	Male −13 Female −5	Male −12 Female −10	0.33
Race	Caucasians −16 Africans −1 Others −1	Caucasians- 15 Africans-6 Others-1	0.12
Blood pressure (mm Hg) Systolic	130 (122–140)	131 (124–140)	0.85
Blood pressure (mm Hg) Diastolic	67 (62–75)	68 (60–72)	0.76
Hemoglobin(g/L)	110.5 (104–119)	108.5 (102–117)	0.39
Serum ferritin(ug/L)	71 (40–143)	67 (27–100)	0.59
TSAT	17 (14–20)	16.5 (10–20)	0.37
Patients on ESA*	7	6	1.0
Average ESA dose (ug/month)	60(60–80)	80(60–100)	0.20
Serum creatinine(umol/L)	246.5 (206–362)	216.5 (176–351)	0.48
Glomerular filtration rate(ml/min/1.73 m2)	20.5 (12–26)	23 (18–33)	0.39
Serum albumin	38 (35–39)	38.5 (36–41)	0.58
Intact PTH	13.05 (6.1-20.1)	13.3 (7.9-16.75)	0.93
Serum phosphate	1.28 (1.07-1.42)	1.28 (1–1.54)	0.96
Cause Of ESRD	Diabetes – 6 Ischemic nephropathy-3 Hypertension – 3 Unknown – 3 Others – 3	Diabetes −9 Ischemic nephropathy-6 Hypertension – 1 Unknown – 5 Others – 1	0.22

The baseline hemoglobin was 110.5 g/L {inter quartile range (IQR): 104–119} in the HIP group and 108.5 g/L (IQR:102–117) in the IV iron sucrose group. The baseline serum ferritin was 71 ug/L (IQR: 40–143) in HIP group and 67 ug/L (IQR: 27–100) in IV iron sucrose group. Baseline serum TSAT was 17% (IQR14-20) in HIP group and 16.5% (IQR 10–20) in IV iron sucrose group. The eGFR was comparable between both groups {20.5 ml/min/1.73 m^2^ (IQR:12–26) in HIP and 23 ml/min/1.73 m^2^ (IQR:18–33) in IV iron sucrose group}. Similar numbers of patients who were already on ESA treatment. Fourteen of eighteen (78%) patients completed the study as per protocol in the HIP group and 19 of 22 (86%) in the IV iron sucrose group.

After 6 months of treatment there were increases in the Hb, TSAT and serum ferritin in both the groups compared to their baseline values (Table 2). There was no difference in the Hb in the HIP group was 117 g/L and 113 g/L in the IV sucrose group at 6 months (p = 0.37; Table 3). Among iron indices, the TSAT at 6 months was also similar in both the groups {21.5%(17–29) in HIP and 21.5% (17–27) in IV sucrose; p = 0.82}, whereas serum ferritin was significantly higher in the IV iron sucrose group compared to HIP group {85.5 ug/L (44–104) in HIP and 244 ug/L (71.5-298); p = 0.004}. In the oral HIP group, in addition to the 6 patients who were on an ESA at study entry, one more patient was started on ESA by study completion. In the IV iron sucrose group, one of the 6 patients was able to discontinue ESA therapy.

**Table 2:** Change in Hgb, ferrtin and TSAT from baseline to 6 months by treatment group

**Parameter**	**HIP baseline**	**HIP 6 m**	**p-value**	**IV iron sucrose baseline**	**IV iron sucrose 6 m**	**p-value**
Hgb	110.5	117	0.15	108.5	113	0.23
	(104–119)	(110–128.8)		(102–117)	(107.5-120.3)	
Ferritin	71	85.5	0.81	67	244	0.003
	(40–143)	(44–104)		(27–100)	(71.5-298)	
TSAT	17	21.5	0.05	16.5	21.5	0.04
	(14–20)	(17–29)		(10–20)	(17–27)	

**Table 3:** Primary and secondary outcomes: hemoglobin, serum ferritin, TSAT and ESA requirement at 6 month

**Parameter**	**HIP**	**IV sucrose**	**p-value**
Hgb (g/L)	117 (110–128.8)	113 (107.5-120.3)	0.37
Serum ferritin (ug/L)	85.5 (44–104)	244 (71.5-298)	0.004
TSAT (%)	21.5 (17–29)	21.5 (17–27)	0.82
Average ESA dose at 6 month (ug/month)	60 (7 patients)	50 (5 patients)	0.56

Three patients in IV iron sucrose and 4 patients in oral HIP group withdrew from the study. In the IV iron sucrose group one patient was non adherent to the study protocol, one patient required blood transfusion and one had surgery requiring withdraw from the study. In HIP group, one patient was non adherent and 3 patients discontinued secondary to new or worsening abdominal cramps. Overall adverse effects are similar between both the groups (Table 4). Four patients in each group had more than one adverse event. Gastrointestinal complaints were the most common adverse effects in both groups with constipation and abdominal cramps being the most common in HIP group and constipation in IV sucrose group. Symptomatic hypotension occurred in 3 patients in the IV iron sucrose group during infusion (13%).

**Table 4:** Adverse events

**Parameter**	**HIP (18)**	**IV sucrose (22)**
> 1 Adverse event	4	4
Constipation	5	4
Diarrhoea	2	3
Bloating sensation	3	2
Abd cramps	5	3
Nausea	2	2
Dyspepsia	1	3
Muscle cramps	5	2
Symptomatic hypotension	0	3
Skin rash	1	0
Overall	28	26

## Discussion

In our single blinded study comparing 11 mg 3 times per day oral HIP to 200 mg IV iron sucrose monthly, we did not find any difference in Hb after 6 months of therapy. The TSAT improved in both groups and was not statistically different between the patients treated with HIP or IV iron sucrose. However, the ferritin increased more in the IV iron sucrose group and this was statistically significant. Adverse events were similar in both groups.

Anemia develops early during CKD and is associated with increased cardiovascular morbidity, mortality and decreased quality of life for CKD patients [1-3]. As CKD progresses, Hgb falls because of a decrease in erythropoietin production as well as iron deficiency that develops secondary to decreased absorption and increased loss. Although iron stores can be restored with either intravenous or oral iron, both therapies have potential risks and benefits. Intravenous iron may be convenient for some patients (especially those on hemodialysis) and may be associated with less gastrointestinal side effects [11,12]. However, intravenous iron is expensive and may be associated with hypotension, serum sickness type reactions and anaphylaxis [13,14]. Oral iron may be more convenient for ND-CKD and peritoneal dialysis patients, but data on the efficacy of oral iron is conflicting.

HIP is produced by hydrolysis of bovine hemoglobin resulting in a highly soluble heme moiety that contains more than 1% iron. Since heme is absorbed via a different receptor than non heme (ionic) iron, the absorption kinetics and gastrointestinal side effect profiles of HIP and ionic iron are dissimilar [29,30]. Administration of HIP to 14 healthy subjects was associated with fewer side effects and significantly higher bioavailability compared with nonheme iron [31]. HIP increased serum iron levels 23 times greater than ferrous fumarate on a milligram-per- milligram basis [31]. Hallberg et al. has also shown enhanced absorption of heme iron compared to iron salts even in subjects with serum ferritin levels greater than 400 ng/mL (898 pmol/L) [33].

Although we did not compare HIP to another non-heme iron, we were able to show that supplementation of HIP to patients with ND-CKD was able to maintain Hb and improve measures of iron indices over a 6 month period. The gastrointestinal adverse events were not greater in the HIP group than the IV iron sucrose group. Our study results are consistent with a study published by Nissenson et al. on hemodialysis patients [34]. They performed an open-label, pre-test/post-test trial of HIP (1 tablet tid) administered instead of intravenous iron to 37 ESA-treated hemodialysis patients over a 6 month period. Although in their study 25% of patients dropped out or were excluded, oral HIP was able to successfully replace IV iron therapy in the majority of patients on hemodialysis. Hematocrit targets and iron stores were maintained and a significant improvement in ESA efficiency (p = 0.04) was reported. However, the results of Nissenson et al. study were limited by the study design, high drop-out rate (25% over 6 months) and failure to analyze on an intention to treat basis [34].

In ND-CKD anemia studies, 7 randomized controlled trials comparing the efficacy of IV iron to oral iron have been reported and yielded contradictory results [24-28,35,36]. The studies differed in several important ways including baseline Hb levels, study duration, iron status of the patients, sample size and type of IV iron preparations. In the meta-analysis by Rozen-Zvi et al., there was a small improvement in Hb concentration in patients treated with IV iron compared to oral iron [0.31 g/dl (0.09 to 0.53)], the clinical significance of this small difference is questionable [37].

In our study HIP, was compared with IV iron sucrose at doses that were considered roughly equivalent over 6 month duration. Under these conditions, HIP appeared to have similar efficacy in maintaining hemoglobin with no increase in gastrointestinal side effects. However, similar to previous randomized studies, the serum ferritin was significantly higher in IV iron group, in spite of similar TSATs in the HIP group. A similar result was seen in the recently completed HEMATOCRIT trial in which the serum ferritin was also higher in peritoneal dialysis patients treated with ferrous sulfate compared to HIP [38]. It is unclear if the increased ferritin is clinically significant. However, the ability to withdraw the ESA in one patient in the IV iron sucrose group but not in the HIP iron group requires further study.

There are several limitations to our study. We had limited ability to detect a difference in Hgb values due to our small sample size (power 0.56). In designing a non-inferiority trial of HIP versus IV iron sucrose with a difference of 2 g/L in the mean of the Hb values would require 694 patients. The difficulties with recruitment and the lack of interest in participation in the study suggest that a repeat study aiming for a larger ‘N’ would not be feasible at our centre. We also did not examine the potential effects on oxidative stress between the two different types of iron and the effect of iron preparations on eGFR [14]. Since the maximum follow up has been 6 months in all the studies including our study, it limits our ability to draw conclusions regarding the long term consequences of different treatment regimens on Hb levels, progression of CKD (eGFR) and clinical outcomes, such as mortality, cardiovascular outcomes, and quality of life. Long term follow-up is especially important given the concerns about oxidative stress, infection risk, and cardiovascular morbidity and mortality secondary to the free iron released into the circulation from the IV preparation [12,15-18]. The ongoing randomized study by Agarwal et al., in which the effect of oral versus IV iron on GFR and proteinuria will be assessed over a 2 year period may address a couple of the issues [39].

## Conclusions

In conclusion, we have shown in this single blinded randomized controlled trial that HIP was as effective in maintaining Hb concentration in ND-CKD patients as IV iron sucrose over a 6 month period. The results of the ongoing randomized studies with longer follow up are required to answer the important questions related to morbidity and mortality. Further studies are also required to determine the optimal time to intervene with iron therapy since iron also has other physiologic functions.

## Competing interests

None of the authors have any conflicts of interest to declare with respect to this study. The results of this study have not been published elsewhere except in abstract form.

## Authors’ contributions

Study design and implementation – AC, JLD, DLZ. Data Analysis –. SPN, AA. Manuscript Preparation – APN, DLZ. All authors review and acceptance of final version of the manuscript.

## Pre-publication history

The pre-publication history for this paper can be accessed here:

http://www.biomedcentral.com/1471-2369/14/64/prepub
